# Electrochemical modulation of host-microbe dynamics in wound healing

**DOI:** 10.3389/fmicb.2026.1796714

**Published:** 2026-04-24

**Authors:** Pramod Bhasme, Surabhi Singh, Shomita S. Mathew Steiner, Sashwati Roy, Chandan K. Sen

**Affiliations:** Department of Surgery, School of Medicine, McGowan Institute for Regenerative Medicine (MIRM), University of Pittsburgh, Pittsburgh, PA, United States

**Keywords:** biofilm-dispersion, electroceuticals, host–microbe interactions, immunomodulation, wound-healing

## Abstract

Wound healing emerges from a tightly orchestrated bioelectric landscape shaped by ion gradients, membrane potentials, and redox dynamics physical cues that direct cell migration, immune activation, and epithelial organization long before biochemical gradients take form. Recent advances reveal that electrical signals constitute a master regulatory layer: Transient receptor potential (TRP)-channel mediated ion flux governs early wound polarity; endogenous transepithelial potential collapse triggers rapid electric fields that guide keratinocyte and fibroblast migration; and connexin-dependent gap-junction coupling coordinates tissue-level responses across multicellular sheets. Electroceutical strategies exploit these principles by recalibrating electrical and electrochemical environments rather than targeting single molecules. This shift enables simultaneous modulation of ion-channel gating, cytoskeletal dynamics, growth-factor signaling, and immunometabolic programs reshaping whole-tissue behavior in ways unattainable with classical pharmacology. Key breakthroughs demonstrate that controlled electrical stimulation can reprogram human macrophages toward reparative phenotypes, enhance keratinocyte electrotaxis even under diabetic conditions, accelerate fibroblast-driven matrix assembly, and amplify endothelial angiogenic responses. Microbial communities respond in the opposite direction. Biofilms, long considered antibiotic-impervious, depend on exquisitely tuned membrane potential, proton motive force, and redox stratification for cohesion and persistence. Low-intensity electrical cues disrupt this energetics, collapsing efflux pump function, silencing quorum systems, loosening EPS architecture, and destabilizing metabolic heterogeneity effects impossible to escape through single gene mutation. Overall, these discoveries frame electroceuticals as system-level disruptors of microbial order and restorers of host coordination. With the emergence of AI-enabled, closed-loop bioelectronic dressings capable of sensing and responding to wound physiology in real time, electricity is poised to become a foundational operating principle for next-generation regenerative and anti-infective therapy.

## 1 Introduction

Wound healing unfolds within a dynamic biophysical environment shaped by ion gradients, membrane potentials, and redox balance, all of which influence how cells migrate, communicate, and organize into functional tissue (Balasubramanian et al., 2024; Bi et al., 2025). In intact skin, coordinated ion transport maintains a stable transepithelial potential that supports epithelial polarity, barrier integrity, and tissue homeostasis (Zhao et al., 2006, 2022). When injury disrupts this structure, the resulting collapse of electrical gradients generates endogenous electric fields that arise well before most biochemical signals associated with inflammation and repair (Borah et al., 2025). Growing evidence indicates that these early bioelectric shifts guide keratinocyte and fibroblast migration, regulate inflammatory responses, and alter local metabolic activity during the initial phases of tissue repair.

Electrical and electrochemical forces also shape microbial behavior. Bacterial cells depend on membrane potential and proton motive force to sustain ATP production, nutrient transport, motility, and coordinated community functions such as biofilm formation (Biquet-Bisquert et al., 2024). Changes in pH, oxygen tension, ionic composition, and redox state within wounded tissue can therefore influence host and microbial processes simultaneously. In chronic wounds, persistent inflammation and tissue degradation further destabilize these electrochemical conditions, hindering tissue regeneration while promoting microbial persistence and biofilm stability (Dai and Chen, 2025). These shared dependencies highlight the bioelectric wound environment as a critical interface linking host tissue responses with microbial physiology.

Electroceutical strategies seek to influence this interface by modulating electrical and electrochemical states rather than targeting individual molecular pathways. Controlled electrical stimulation has been shown to alter ion-channel activity, cytoskeletal organization, intracellular signaling networks, and immunometabolic states across multiple cell types (Evans and Sen, 2023). Because electrical modulation acts on upstream physical cues, its effects can propagate across molecular, cellular, and tissue scales simultaneously. This systems-level influence differs from conventional pharmacologic approaches, which typically rely on specific ligand target interactions (Chen et al., 2022). In complex wound environments where signaling pathways overlap and vary spatially, modifying the underlying biophysical landscape may enable more coordinated and integrated biological responses than single-target therapeutic strategies alone (Tripathi et al., 2022).

Electrical cues regulate migration, adhesion dynamics, and metabolic activity at the cellular level, processes essential for epithelial closure and stromal remodeling. At the tissue level, bioelectric signals can promote coordinated responses among cell populations through gap-junction connectivity and extracellular field effects (Shim et al., 2024). Similar perturbations can also influence microbial systems. Membrane potential and proton gradients represent central components of microbial physiology, and disturbances in these parameters can impair microbial energetics, nutrient transport, quorum sensing, and biofilm cohesion (Ungureanu et al., 2025; Akabuogu et al., 2024). Because these effects arise from fundamental physiological constraints rather than inhibition of individual molecular targets, microbial adaptation may be more limited compared with conventional antimicrobial therapies.

Electrical modulation therefore affects wound environments via two complementary mechanisms: (1) enhancing coordinated host repair, and (2) destabilizing microbial survival strategies. This dual influence is particularly relevant in chronic wounds, where impaired healing and persistent infection coexist and reinforce one another. By acting on electrochemical conditions that shape both host tissues and microbial communities, electroceutical interventions can promote tissue regeneration while reducing factors that sustain infection. This review examines electrochemical modulation across molecular, cellular, microbial, and clinical contexts and highlights emerging adaptive electroceutical technologies.

## 2 Foundations of electrical and electrochemical regulation in living systems

### 2.1 Ion flux, membrane potential, and tissue-level coordination

**Figure 1:**
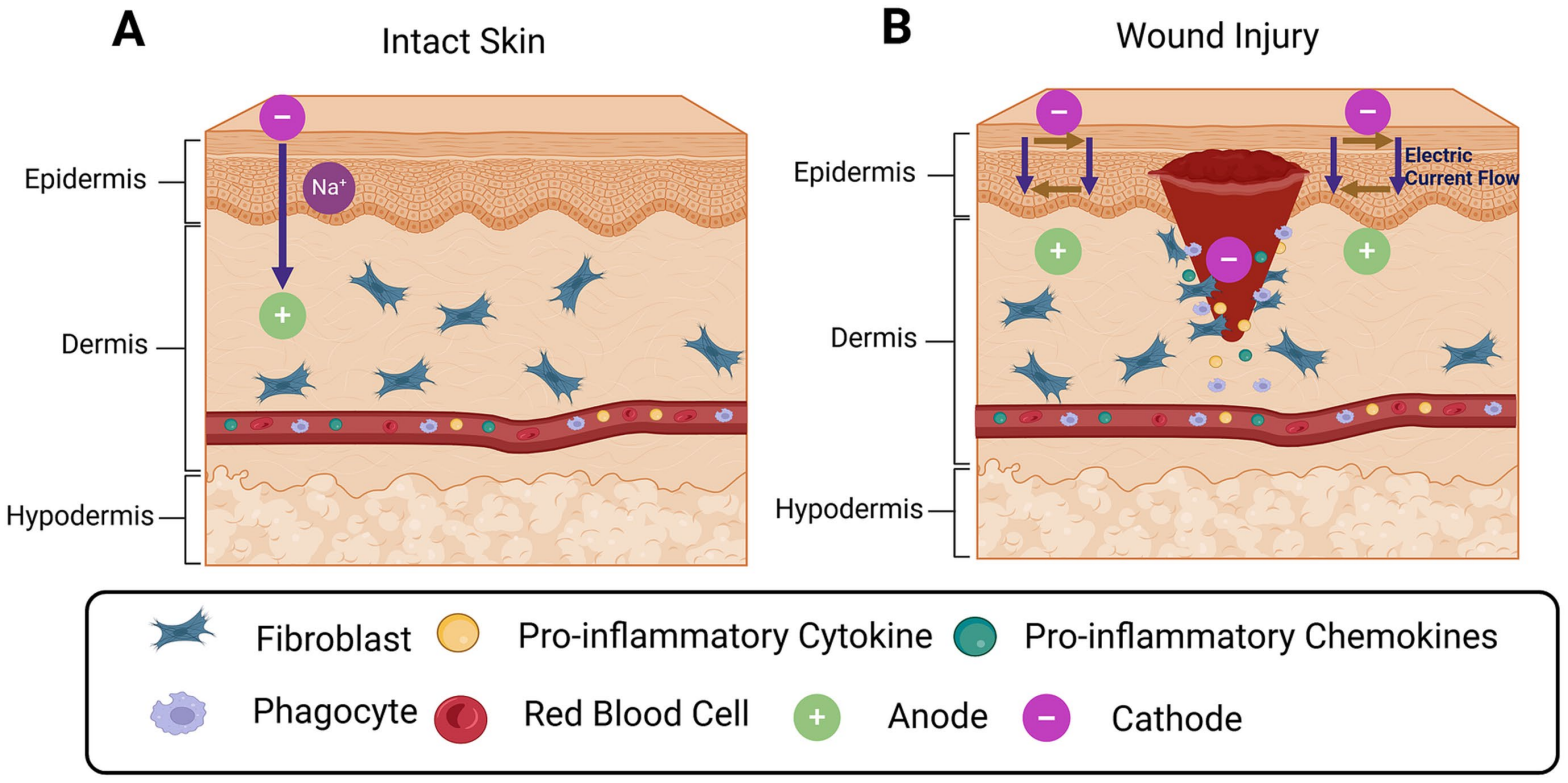
Endogenous bioelectric architecture of intact skin and injury-induced electrical signaling: **(A)** Intact epidermis maintains a stable transepithelial potential through polarized ion transport across epithelial layers, supporting tissue homeostasis. **(B)** Disruption of epithelial integrity collapses this potential and generates endogenous lateral electric fields and current flow toward the wound bed, providing early directional cues that guide keratinocyte and fibroblast migration and initiate coordinated inflammatory and repair responses. Created in BioRender. Bhasme, P. (2026). https://BioRender.com/fb7rmrf.

Imagine the moment a piece of skin is breached. Before immune cells mobilize, before cytokines accumulate, and long before new tissue fills the void, the wound undergoes a dramatic electrical event a collapse of its transepithelial voltage that instantly reshapes ion flow across the injured boundary. This electrical shift is not merely a byproduct of injury but an active organizer of the healing response (Zhao et al., 2006, 2022; Borah et al., 2025). This injury-induced transition from a polarized epithelial barrier to a wound-centered electrical sink establishes the earliest physical signals that organize subsequent cellular and immune responses (Figure 1). As illustrated in Figure 1, intact skin maintains a stable transepithelial potential through coordinated ion transport, whereas disruption of epithelial continuity collapses this polarity and generates lateral electric fields that drive directional ion flow, fibroblast migration, and immune cell recruitment toward the wound bed.

Biological systems operate within finely tuned electrical and electrochemical environments that shape cell behavior, tissue organization, and host–microbe interactions (Sani et al., 2023). These endogenous bioelectric cues function as a foundational regulatory layer that constrains how biochemical and mechanical signals are interpreted rather than acting as isolated stimuli. Injury, infection, or sustained inflammation disrupts this layer, resulting in loss of coordinated cellular responses and persistence of pathological states. Electroceutical strategies seek to restore or recalibrate these disrupted physical signals, positioning intervention upstream of conventional molecular signaling pathways (Zhang et al., 2025).

Over the past few years, researchers have looked deeper into how ion flux and membrane potential function as early navigators in wound repair. Transient receptor potential (TRP) channel-focused reviews from 2023–2024 highlight that the movement of Na^+^, K^+^, Ca^2+^, and Cl^−^ across the epithelial membrane modifies the local transmembrane potential, which then feeds into changes in the transepithelial potential (TEP) surrounding a lesion (Elliott and Cooper, 2024). The decline in TEP produces a directional electrical gradient that keratinocytes, fibroblasts, and immune cells can interpret as a map guiding them toward the wound center (Tavares-Ferreira et al., 2025). Coordinated ion transport across intact epithelial layers establishes stable transepithelial potential under homeostatic conditions. Upon injury, collapse of this organization generates lateral electric fields oriented toward the wound bed, which act as instructive signals rather than passive consequences of tissue damage (Borah et al., 2025). These endogenous wound electric fields influence cell polarity, cytoskeletal organization, and migratory directionality well before chemokine gradients emerge.

#### 2.1.1 Coordinated ion dynamics in wound bioelectricity

Coordinated ion transport across epithelial membranes establishes spatially organized membrane potential that acts as instructive signals during tissue repair (Zhao et al., 2006, 2022). Injury disrupts these gradients, initiating rapid electrical and biochemical responses that shape cellular behavior (Zhao et al., 2006, 2022). TRPV1, TRPV3, TRPV4, TRPA1, and other TRP channels serve as sensors of mechanical stress, inflammation, and local ionic imbalance, modifying membrane potential in ways that tune activation thresholds (Liao et al., 2026). TRP channel activation modulates re-epithelialization, while suppression of channels such as TRPM8 accelerates wound closure, highlighting that ion channels influence tissue-level behavior rather than functioning solely as ion conduits (Katoh, 2023).

**Figure 2:**
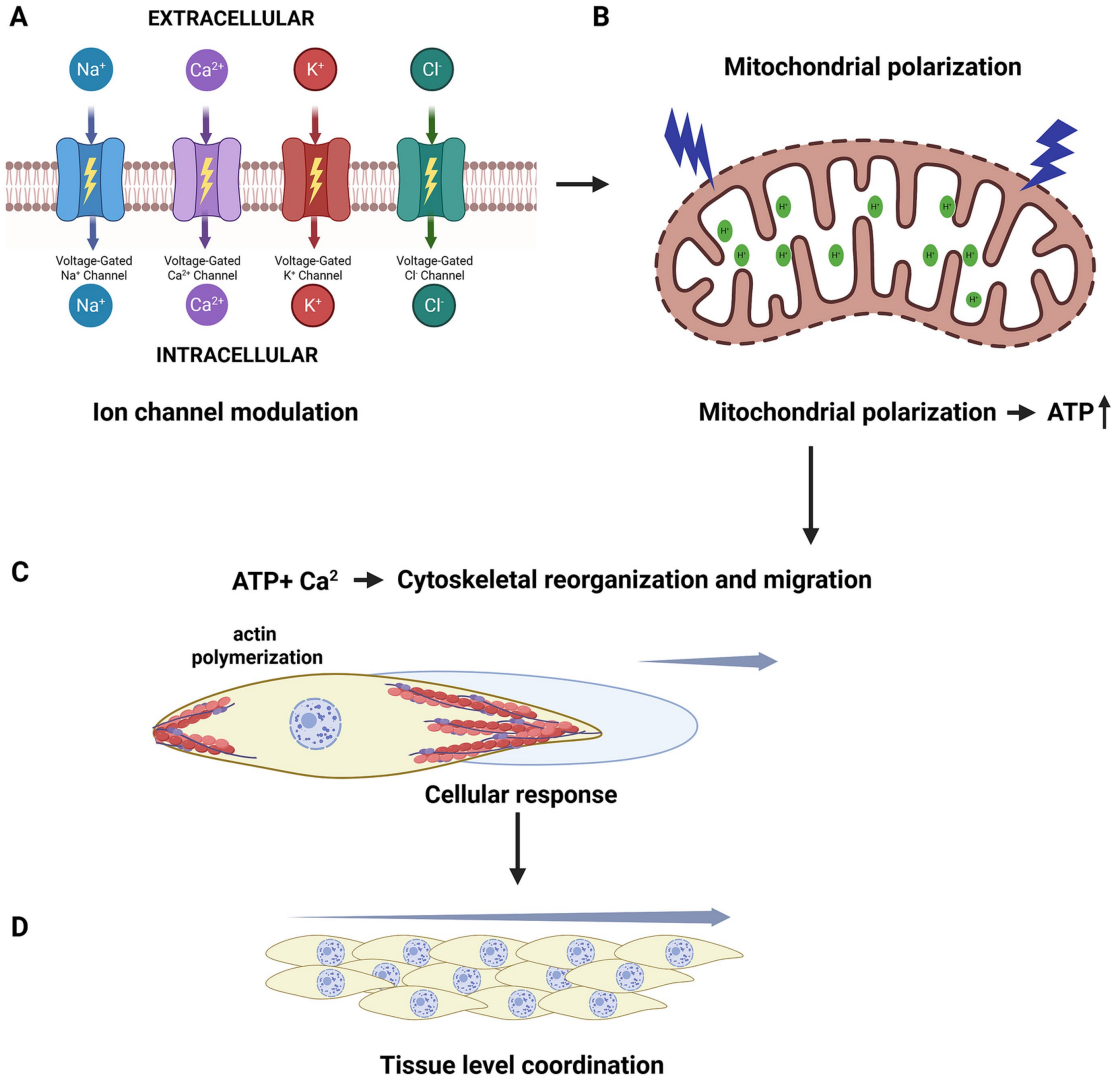
Electroceutical mechanisms across molecular to tissue scales: **(A)** Electric fields modulate voltage-gated Na^+^, Ca^2+^, K^+^, and Cl^−^ channels, altering membrane polarization and intracellular ion fluxes. **(B)** Ion flux driven changes in mitochondrial membrane potential enhance proton motive force and ATP production. **(C)** Elevated ATP and Ca^2+^ promote actin polymerization, cytoskeletal remodeling, and directional cell migration. **(D)** At the tissue scale, electrically guided cells align along field vectors, producing coordinated epithelial and stromal movement that drives wound closure. Created in BioRender. Bhasme, P. (2026). https://BioRender.com/fb7rmrf.

At the subcellular level, membrane polarization regulates channel gating, transporter kinetics, and enzyme activity across plasma and organellar membranes (Elliott and Cooper, 2024). These electrical shifts alter activation thresholds and signaling kinetics, enabling cells to integrate electrical, biochemical, and mechanical cues in a graded manner (Ridgway-Brown et al., 2025). Modest changes in membrane potential can therefore produce large tissue-scale effects without inducing cytotoxicity (Ridgway-Brown et al., 2025). The resulting intracellular signals propagate through mitochondrial polarization, cytoskeletal remodeling, and metabolic reprogramming, collectively shaping multicellular wound responses (Figure 2).

#### 2.1.2 Electric fields as early patterning cues

A microfluidic electrotaxis study revealed that sustained endogenous electrical cues outperform biochemical cues when guiding keratinocyte sheet migration. Uni-directional electrical stimulation increased wound closure rates nearly three-fold even restoring the impaired motility of diabetic keratinocytes to near-normal levels. These findings highlight how electrical cues act as patterning signals that sculpt multicellular organization long before growth factors or cytokines dominate the scene (Liu et al., 2025). Keratinocytes and fibroblasts migrate directionally in response to voltage gradients through polarized redistribution of growth-factor receptors, actin remodeling, and focal-adhesion turnover (Zhang et al., 2025). Importantly, bioelectric guidance precedes chemokine-driven migration and remains effective even when biochemical gradients are disrupted, establishing early spatial organization of the wound environment (Zhu et al., 2025).

#### 2.1.3 Gap junctions as the tissue’s communication grid

As electricity flows through wounded epithelium, gap junction proteins particularly connexins play a crucial role in distributing ions and second messengers (Lukowicz-Bedford et al., 2024). These junctions allow neighboring cells to synchronize membrane potential oscillations, calcium waves, and metabolic states (Cervera et al., 2023). The result is collective decision-making: cells “know” which direction to move, when to proliferate, and when to pause. Gap junction mediated coupling enables epithelial sheets to function as coherent bioelectric units by permitting rapid transmission of ions and small metabolites across connected cells (Lukowicz-Bedford et al., 2024). Perturbation of transepithelial potential generates extracellular electric fields that propagate laterally beyond the site of injury, biasing membrane polarization and ion-channel activity in neighboring cells (Tavares-Ferreira et al., 2025). Synchronization of intracellular calcium oscillations provides a shared temporal framework that aligns cellular responses without enforcing uniform molecular activation (Cervera et al., 2023). Newer studies of epithelial sheet behavior under controlled electric fields (2023–2024) confirm that disrupting gap junction communication leads to loss of directional coherence, even when electrical cues remain intact. This reinforces the emerging idea that ion flux and tissue connectivity operate together to coordinate spatial signaling and intercellular communication.

#### 2.1.4 The injury triggered electrical cascade

In many respects, the electrical collapse at the wound edge represents the earliest physiological response to tissue injury (Sun et al., 2023). Disruption of epithelial integrity rapidly dissipates the transepithelial potential and generates lateral endogenous electric fields across the wound bed, transforming a polarized epithelial barrier into an electrically active injury microenvironment (Borah et al., 2025; Tavares-Ferreira et al., 2025). These bioelectrical changes arise immediately following Tissue disruption and precede classical biochemical signaling pathways that govern inflammation and repair. This injury-induced electrical shift instantly establishes several coordinated processes. First, the collapse of epithelial polarity creates a directional electric field extending across the wound site, providing spatial guidance cues that influence cell migration and tissue organization (Tavares-Ferreira et al., 2025). Second, rapid ion fluxes involving sodium, potassium, calcium, and chloride modify ion-channel activity and intracellular signaling states, altering cellular responsiveness to injury-associated stimuli (Elliott and Cooper, 2024). Third, changes in membrane potential sensitize cells to mechanical and biochemical signals by shifting activation thresholds and modulating cytoskeletal dynamics and signaling pathways (Ridgway-Brown et al., 2025). Fourth, synchronized tissue responses emerge through gap-junction connectivity, enabling propagation of ions, metabolites, and calcium signals across multicellular domains and promoting coordinated adaptation within epithelial sheets (Lukowicz-Bedford et al., 2024; Cervera et al., 2023). These events occur within seconds to minutes, far earlier than transcriptional responses or immune-cell infiltration, underscoring their role as proximal regulators of the wound response (Belliveau et al., 2025; Fernández-Yagüe et al., 2025).

### 2.2 Electrochemical forces governing microbial physiology

**Figure 3:**
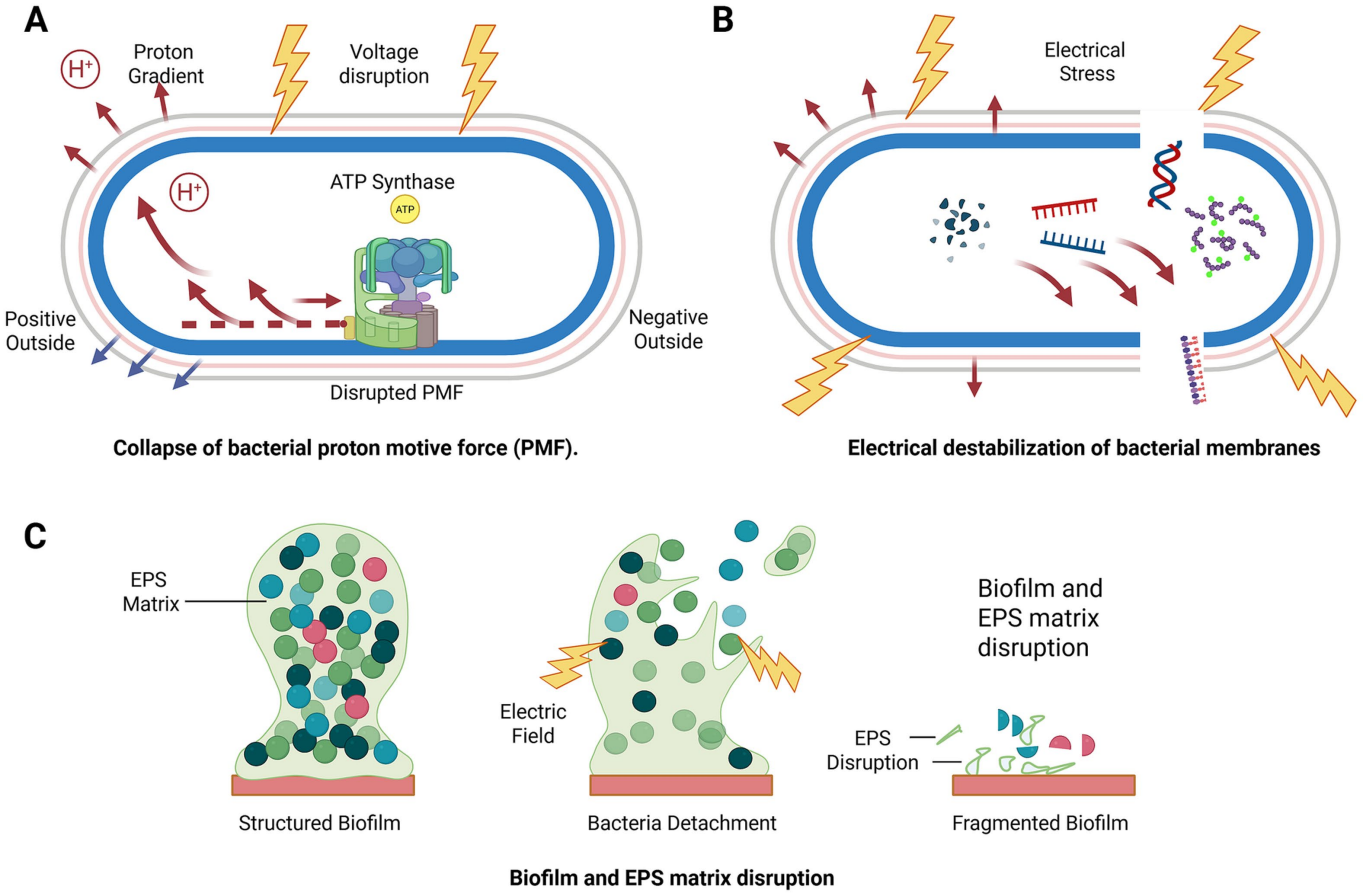
Electrochemical disruption of microbial biofilms. **(A)** Electric fields dissipate bacterial proton motive force and disrupt bioenergetic coordination. **(B)** Electrical perturbation alters membrane energetics and transporter function, leading to functional leakage and impaired tolerance without overt membrane rupture. **(C)** Electrochemical perturbation weakens charge-dependent interactions within the extracellular polymeric substance (EPS), destabilizing biofilm architecture and promoting fragmentation and cell detachment. Created in BioRender. Bhasme, P. (2026). https://BioRender.com/fb7rmrf.

If ion flux and membrane potential set the stage for host repair, they are nothing short of existential for microbes. In the microbial world, survival depends on maintaining tightly regulated electrochemical balance. Every bacterium lives by maintaining a delicate membrane potential and proton motive force that fuels ATP synthesis, nutrient acquisition, motility, and the chemical “conversation” that lets neighboring cells coordinate behavior (de la Viuda et al., 2025). Unlike mammalian cells, microbes operate with minimal redundancy in maintaining electrochemical stability. They lack mitochondrial buffering and rely largely on surface-level energetics, making even modest electrical perturbations physiologically destabilizing (Lee et al., 2024). This asymmetry forms the basis of electroceutical selectivity. Electrical perturbation destabilizes these constraints by collapsing proton motive force, disrupting membrane energetics, and weakening biofilm architecture, thereby undermining microbial persistence (Figure 3). Key bioelectric and electrochemical mechanisms operating across molecular, cellular, and tissue scales are summarized in Table 1. The table integrates how electrical cues regulate ion-channel activity, mitochondrial energetics, redox balance, cytoskeletal dynamics, and collective cell behavior, linking these processes to functional outcomes in host repair and microbial destabilization. By organizing these mechanisms across biological scales, the table provides a unifying framework that connects foundational bioelectric principles with downstream effects on wound healing and infection control.

**Table 1:** Mechanistic actions of electroceuticals across biological scales relevant to infection control and tissue repair.

Scale	Biological target	Electroceutical mechanism	Functional outcome	References
Molecular	Voltage-gated ion channels	Electric fields alter channel gating probability and activation thresholds	Tuned excitability and signal initiation	Huang et al. (2023)
Molecular	Proton gradients	Modulation of transmembrane proton flux affects bioenergetic balance	Stabilized intracellular pH and metabolism	Biquet-Bisquert et al. (2024)
Molecular	MAPK/ERK signaling (field driven receptor redistribution)	Redistribution of membrane receptors under electrical cues	Enhanced migration-associated signaling	Fei and Guo (2025)
Subcellular	Mitochondrial membrane potential	Electrical perturbation influences ΔΨm and ETC efficiency	Optimized ATP output, reduced oxidative stress	Preetam et al. (2024)
Subcellular	ROS signaling microdomains	Electric fields reshape redox gradients	Controlled ROS signaling without cytotoxicity	Hunt et al. (2024)
Subcellular	Cytoskeletal organization	Electrical bias alters actin polymerization and focal adhesion turnover	Directional force generation	Fernández-Yagüe et al. (2025)
Cellular (Epithelial)	Keratinocyte migration	Endogenous wound electric fields guide directional motility	Accelerated re-epithelialization	Zhang et al. (2025)
Cellular (Immune)	Macrophage polarization	Bioelectric cues alter immunometabolic state	Shift toward pro-resolution phenotypes	Bianconi et al. (2024)
Cellular (Immune)	Neutrophil chemotaxis	Electric fields guide directional immune cell migration	Improved targeting and clearance	Belliveau et al. (2025)
Cellular (Immune)	Calcium signaling	Electrical stimulation synchronizes Ca^2+^ oscillations	Coordinated immune activation	Bi et al. (2025)

#### 2.2.1 The microbial battery that cannot fail

In the last few years, studies of bacterial electrophysiology have revealed that microbial physiology is strongly governed by membrane electrochemical gradients: their membrane potential is not a background variable, it is their operating system. A 2024 study showed that biofilms actively shape and modulate proton motive force across their spatial layers, creating pockets of high and low energetic activity that support division of labor within microbial communities (Biquet-Bisquert et al., 2024). These electrochemical gradients underpin core processes ranging from ATP synthesis to nutrient uptake and signaling coordination (Akabuogu et al., 2024). But microbes carry a vulnerability: unlike mammalian cells that have layered redundancies to maintain electrical stability, bacteria operate with leaner systems. They lack mitochondrial buffering and rely largely on surface-level energetics, making them exquisitely sensitive to even modest electrical disruption. This asymmetry forms the basis of many new therapeutic strategies (Akabuogu et al., 2024).

#### 2.2.2 Biofilms: electrically stratified cities with hidden power lines

Recent biofilm research has uncovered the remarkable complexity of these structures. They are not random microbial clusters but electrostatically stabilized communities, attached together by extracellular DNA, charged polysaccharides, and protein scaffolding (Hindieh et al., 2025). These components interact like the reinforcing rods of concrete maintaining cohesion and shaping the matrix’s porosity. Electrostatic interactions within the extracellular matrix influence transport behavior and antimicrobial susceptibility in biofilm communities, where electrochemical properties contribute to structural stability and resistance to external stresses (Akabuogu et al., 2024; Azeem et al., 2025). The result is metabolic stratification: energetic outer layers, shielded inner sanctums, and a spectrum of physiological states that thwart immune clearance (Adekoya et al., 2025). Recent reviews emphasize that redox gradients play a dual role inside biofilms: They enable metabolic specialization, allowing bacteria in deeper layers to slow growth and evade antibiotics. They stabilize the extracellular polymeric substance (EPS) by maintaining charge interactions essential for structural integrity. This means biofilms do not just resist treatment they engineer their own electrochemical resilience (Hindieh et al., 2025).

#### 2.2.3 When electricity turns against the microbe

Over the past 3–5 years, a growing body of work has shown how electrical and electrochemical disturbances can unravel microbial defenses. Even weak external electrical fields, far below electroporation thresholds, can partially depolarize microbial membranes, short circuiting the machinery required for ATP production, nutrient transport, and stress tolerance (Whittle et al., 2024; Zhu et al., 2024). These effects arise from disruption of fundamental electrochemical constraints rather than direct membrane damage, distinguishing electroceutical action from classical bactericidal mechanisms (Lee et al., 2024). A 2024 study revealed that subtle shifts in redox balance and proton gradients significantly disrupt membrane-bound enzymes, impairing energy generation and compromising efflux pumps central to antibiotic tolerance. Because respiratory coupling, transporter activity, and efflux pump function depend directly on membrane potential and proton motive force, even modest depolarization propagates system-wide dysfunction across microbial populations rather than remaining confined to individual molecular targets (Whittle et al., 2024; Juncker et al., 2022). Once membrane potential falters, downstream consequences cascade through the bacterial collective:

##### 2.2.3.1 Bioenergetic and enzymatic dysfunction following membrane depolarization

Membrane depolarization disrupts proton motive force dependent respiration, impairing ATP synthase activity and electron transport chain efficiency (Gerry et al., 2025). Because many bacterial enzymes rely on electrochemical gradients for optimal kinetics, shifts in membrane potential alter catalytic rates without requiring direct molecular inhibition. Depolarization also compromises ion-coupled transport systems including nutrient suppliers and efflux pumps reducing nutrient acquisition while weakening antimicrobial extrusion capacity (Whittle et al., 2024). These metabolic disturbances perturb NADH/NAD^+^ balance and redox cycle, generating oxidative stress and diminishing metabolic flexibility required for adaptive responses to environmental stress (Amjad et al., 2021). Within biofilms, energetic perturbation disrupts metabolic stratification and cooperative division of labor, weakening coordinated physiological states that normally support persistence and tolerance (Kalashnikova et al., 2023; Roothans et al., 2025). The cumulative outcome is a distributed bioenergetic crisis characterized by reduced ATP availability, impaired transport, redox imbalance, and loss of collective metabolic resilience across microbial populations (Mohiuddin et al., 2024).

##### 2.2.3.2 Quorum sensing networks shut down

Quorum sensing depends on stable membrane energetics and metabolic balance to sustain signal production, release, and detection across microbial populations (Chu and Yang, 2024). Many quorum molecules are synthesized through energy-intensive pathways that require adequate ATP and redox cofactors, while receptor activation and downstream gene expression are sensitive to the physiological state of the cell. When membrane potential is perturbed, these processes become less efficient even without direct interference with signaling proteins. Electrical disruption can impair proton motive force-dependent transport involved in autoinducer secretion and uptake, while shifts in membrane potential and intracellular redox state alter signaling thresholds and transcriptional responses (D'Aquila et al., 2024; Mitra, 2024).

These changes translate into weakened population-level coordination. In *Pseudomonas aeruginosa*, altered energetics interferes with Las, Rhl, and PQS signaling networks, reducing the production of virulence factors and matrix components that normally support biofilm maturation (García-Diéguez et al., 2023). In *Staphylococcus aureus*, disruption of membrane energetics perturbs Agr signaling, diminishing coordinated toxin expression and collective behavior (Eltaher et al., 2026). Because quorum sensing regulates extracellular matrix production, stress responses, and metabolic cooperation, its destabilization reduces biofilm cohesion and adaptive capacity. Rather than targeting specific signaling pathways, electrical perturbation alters the energetic context required for microbial communication. As coordination breaks down, structured communities transition into less organized populations with reduced virulence and persistence potential, increasing their susceptibility to immune clearance and antimicrobial intervention (Zhu et al., 2023; Mitra, 2024).

##### 2.2.3.3 Biofilm architecture destabilizes

Electrostatic bonds within the extracellular polymeric substance (EPS) loosen, making the structure more permeable and vulnerable to immune and antimicrobial attack.

Disruption of redox- and charge-dependent interactions destabilizes extracellular DNA, polysaccharides, and protein scaffolds, collapsing biofilm integrity without requiring enzymatic matrix degradation (Hindieh et al., 2025; Balato et al., 2025). Recent studies indicate that, electrochemical modulation does not kill microbes by force it removes the energy conditions required for them to remain organized. By dismantling population-level electrochemical coordination, electroceuticals convert structured, tolerant communities into metabolically fragile assemblies (Lee et al., 2024).

#### 2.2.4 Why microbes cannot adapt easily

Unlike antibiotic threats that target specific molecules, electrochemical disruption strikes at universal, physical constraints from proton gradients to surface charge interactions. Recent works underscore that these constraints are distributed across multiple interconnected cellular systems rather than encoded by single genes or linear pathways (Juncker et al., 2022; Zhang and Levin, 2025). Because bacteria depend on stable membrane potential and proton gradients for survival, their ability to adapt to electrical perturbation through single-gene mutations is limited. Unlike target-specific antimicrobials, electroceutical interventions impose diffuse physiological stress that is difficult to compensate for through conventional resistance mechanisms. Electrical modulation therefore exploits a fundamental microbial constraint rooted in universal electrochemical requirements. In contrast to target-centric therapies that impose strong selective pressure on individual proteins, electroceutical interventions generate diffuse physiological stress that may be difficult to compensate through mutation or horizontal gene transfer (Lee et al., 2024; Juncker et al., 2022). In this way, electrical intervention exploits a microbial fundamental physiological constraint. Their survival depends on maintaining an electrochemical order they cannot modify without destroying themselves.

#### 2.2.5 A new lens on microbial vulnerability

Overall, recent studies provide increasing evidence that microbial survival depends on maintaining stable electrochemical gradients, which are preserved within structured biofilm matrices characterized by spatial redox heterogeneity. These structured biofilm matrices preserve energetic heterogeneity, metabolic stratification, and collective resilience through finely tuned electrochemical gradients (Whittle et al., 2024; Hindieh et al., 2025). But when external electrical cues shift even subtly, electrochemical disruption undermines the energetic coordination and matrix integrity that sustain biofilms. When external electrical cues shift even modestly, microbial communities lose their structured metabolic organization and become energetically fragile, rendering them more susceptible to immune clearance and antimicrobial agents (Balato et al., 2025). Electroceuticals thus function not as electrical antibiotics but as system-level disruptors of microbial order.

## 3 Electrochemical disruption of microbial survival strategies

### 3.1 Electrochemical destabilization of structurally organized biofilm matrices

Persistent wound infections are rarely driven by lone bacterial wanderers. Instead, they arise from structured biofilms are electrochemical ecosystems organized by ionic, redox, and charge-dependent gradients. These gradients stabilize microbial communities and coordinate metabolic activity. Recent research has reshaped our view of these structures: rather than loose assemblies of cells, they function as electrochemical ecosystems organized by charge, redox, and energetic gradients (Cavallo et al., 2024). Importantly, they also possess exploitable vulnerabilities. Advanced imaging, molecular profiling, and *in situ* electrochemical measurements have revealed that biofilms actively engineer their microenvironment by shaping oxygen tension, pH, ionic composition, and redox state. These physicochemical gradients are not passive consequences of growth but regulated features that stabilize microbial communities and modulate interactions with host tissues (Haval et al., 2025; Zhao et al., 2023; Dai and Chen, 2025). Within chronic wounds, such biofilm-driven environmental remodeling directly impairs keratinocyte migration, macrophage polarization, and angiogenic signaling, reinforcing inflammatory persistence (Li et al., 2021; Al-Taweel et al., 2025). Over the past 3–5 years, studies in microbiology and wound-care science have shown that electrical and electrochemical disturbances can exploit these weak points more effectively than traditional biochemical therapies. These discoveries are reshaping how we understand infection control.

### 3.2 Membrane depolarization and energetic stress: when microbial energetic function is impaired

A microbe’s key vulnerability is the collapse of its membrane potential. This potential is not simply an incidental electrical property it is the essential energetic driver, driving ATP synthesis, nutrient uptake, and motility. Microbial membrane potential and proton motive force function as central hubs linking metabolism, transport, stress tolerance, and signaling. Weak external electric fields well below electroporation thresholds can partially depolarize microbial membranes, disrupting these hubs without causing overt membrane rupture (Zhu et al., 2024; Whittle et al., 2024). Importantly, this mode of disruption targets energetic coordination rather than structural integrity, producing physiological stress that microbes struggle to buffer. Recent work on microbial energetics shows that weak external electric fields can shift membrane potential without causing membrane rupture, leading to measurable perturbations in bioenergetic function. Even modest depolarization can disrupt the proton motive force required for respiration and ATP synthesis, thereby altering energy-dependent cellular processes (Juncker et al., 2022; Zhu et al., 2024). Because multidrug efflux pumps and ion-coupled transport systems depend directly on proton motive force, reductions in membrane potential can impair drug extrusion and increase antimicrobial susceptibility (Juncker et al., 2022; Whittle et al., 2024). Efflux pump activity, respiratory chain efficiency, and ion-coupled transport processes are energetically interdependent. Partial depolarization therefore propagates dysfunction across multiple systems simultaneously, including reduced drug extrusion, impaired nutrient acquisition, and altered stress-response signaling (Juncker et al., 2022; Whittle et al., 2024). Because these effects emerge from distributed energetic collapse rather than inhibition of single proteins, microbes may not readily compensate through pathway redundancy (Lee et al., 2024). This electrochemical disruption produces sublethal energetic stress, enough to weaken microbial defenses but gentle enough to avoid harming host cells.

Low-intensity electrical modulation thus imposes a bioenergetic tax on microbial populations while remaining within tolerable limits for host tissues. This differential sensitivity reflects fundamental asymmetries between microbial and mammalian energy buffering capacity and underlies the therapeutic window exploited by electroceutical approaches (Lee et al., 2024).

Because the intervention targets physical constraints rather than specific proteins, microbes may be less able to escape through single-gene mutations. These dependencies on membrane potential represent fundamental physiological constraints that may limit adaptive escape (Bedendi et al., 2022). In effect, low-intensity electrical modulation turns the microbe’s most fundamental survival strategy its electrochemical battery into its greatest vulnerability (Elbaiomy et al., 2025).

### 3.3 Destabilization of biofilm architecture

Biofilms are complex structural assemblies, built not from stone but from polysaccharides, proteins, and extracellular DNA held together by electrostatic forces. The EPS matrix acts as a charged scaffold that regulates mechanical stability, diffusion of solutes, and spatial organization of metabolic activity within the biofilm. Electrostatic interactions between extracellular DNA, polysaccharides, and proteins create a semi-permeable barrier that both protects resident microbes and enforces metabolic stratification (Hindieh et al., 2025; Sharma et al., 2023).

Charged components of the extracellular polymeric substance including extracellular DNA, polysaccharides, and structural proteins shape mechanical stability, transport behavior, and electrostatic interactions within biofilms (Hindieh et al., 2025). Electrochemical conditions further influence microbial organization and resistance phenotypes within these communities (Akabuogu et al., 2024; Azeem et al., 2025). Electrochemical modulation disrupts these charge-dependent interactions by altering local ionic balance and redox conditions, weakening matrix cohesion without requiring enzymatic degradation. As structural integrity declines, antibiotics penetrate more effectively and immune cells gain access, exposing bacteria previously shielded within the biofilm matrix. Electrochemical interventions exploit this architecture by changing local ionic balance and redox conditions. When pH shifts or redox gradients are disrupted, the charged bonds holding the extracellular polymeric substance begin to loosen.

Electrical modulation induces localized redox reactions and transient pH alterations at the wound surface, destabilizing electrostatic interactions within the EPS matrix and weakening microbial envelopes without requiring enzymatic degradation (Balato et al., 2025). Loss of matrix cohesion disrupts metabolic synchronization and increases susceptibility to both host defenses and adjunctive antimicrobial therapies. As cohesion fades, the matrix integrity is reduced. Antibiotics penetrate more easily, immune cells gain access, and microbes once deeply protected face the full force of host defenses. Electrical modulation does not attack the microbe directly; it disrupts matrix cohesion, leaving pathogens exposed.

### 3.4 Interference with quorum sensing and community co-ordination: silencing the microbial co-ordination

Within biofilms, quorum sensing coordinates collective behaviors such as virulence factor production, matrix synthesis, and stress adaptation, and electrical perturbation can disrupt the electrochemical conditions required for this signaling, thereby weakening coordinated microbial responses (Ungureanu et al., 2025; Akabuogu et al., 2024). Quorum sensing efficacy depends on intact membrane energetics, redox balance, and controlled diffusion of signaling molecules through the biofilm matrix. Electrical perturbation indirectly disrupts these systems by reshaping the electrochemical context required for signal synthesis, transport, and perception rather than by blocking individual receptors (Gonzales et al., 2024). Shifts in membrane potential alter receptor behavior, while changes in redox state interfere with synthesis of quorum molecules. When electrical balance is disturbed, the quorum-sensing communication becomes disrupted. In *Pseudomonas aeruginosa*, electrical modulation interferes with Las, Rhl, and PQS systems, reducing expression of elastases, rhamnolipids, and biofilm maturation genes (Markowska et al., 2024). In *Staphylococcus aureus*, perturbation of membrane-associated Agr signaling suppresses toxin production and destabilizes biofilm organization (Polaske et al., 2023). Without coordinated communication, biofilms lose their ability to behave like an organized collective. They become scattered clusters rather than a unified defense network. Electrical disturbance, in essence, breaks the microbial social contract.

### 3.5 Enhanced susceptibility to antimicrobials and reduced resistance

Recent studies suggest that electrochemical disruption makes antibiotics work better not by boosting the drugs themselves, but by degrading the microbial systems that resist them. Biofilm-associated persistence is driven primarily by phenotypic tolerance rather than stable genetic resistance. Repeated antimicrobial exposure under these conditions promotes resistance selection in surrounding planktonic populations exposed to sublethal drug concentrations (O’Reilly et al., 2025; Sousa et al., 2025). By weakening efflux pumps, destabilizing the matrix, collapsing redox stratification, and interrupting communication, electrical modulation shifts microbial populations into states where antibiotics penetrate more effectively, and immune cells kill more efficiently. Electroceuticals mitigate may help resistance emergence by targeting shared physical constraints membrane potential, proton gradients, redox balance, and electrostatic interactions that are not encoded by single genes or linear pathways (Ungureanu et al., 2025). Distributed physiological stress may limit microbial capacity to adapt through mutation or horizontal gene transfer (Bedendi et al., 2022). Emerging evidence suggests. Electrical modulation disarms microbial defenses in ways antibiotics alone cannot. By weakening microbial persistence without imposing strong target-specific selective pressure, electroceuticals align infection control with long-term antimicrobial resistance mitigation in chronic wound environments (Tran et al., 2024).

## 4 Host responses: electrical modulation of coordinated tissue repair

**Figure 4:**
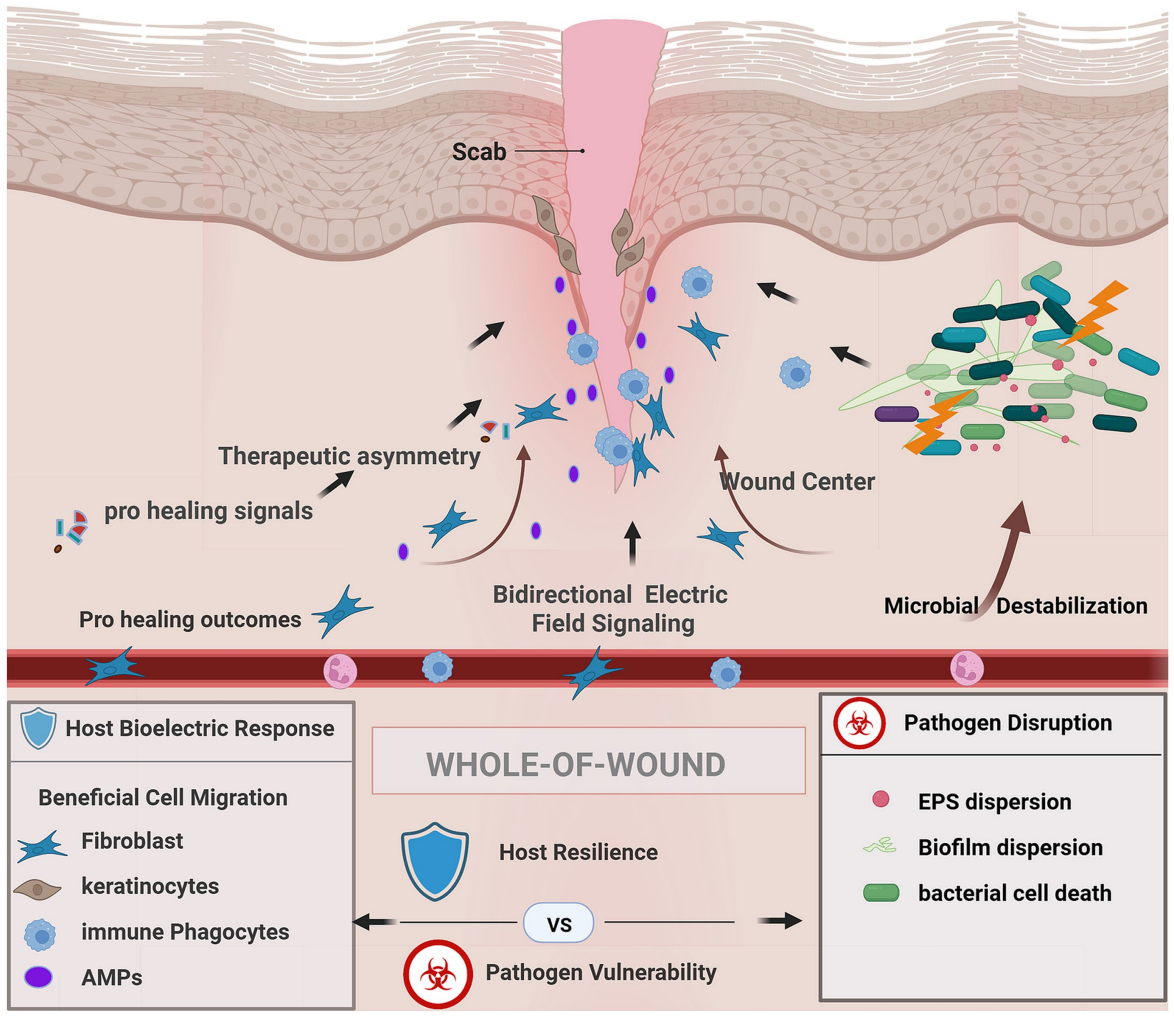
Whole-of-wound bioelectric modulation creates therapeutic asymmetry. Bidirectional electric field signaling coordinates host cell migration and immune responses while destabilizing microbial biofilms through electrochemical stress, promoting host resilience and pathogen vulnerability across the wound bed. Created in BioRender. Bhasme, P. (2026). https://BioRender.com/fb7rmrf.

Wounds do not heal because cells respond to signaling cues they heal because cells interpret signals. And among the earliest and most powerful signals are electrical cues. Over the last 3–5 years, researchers have uncovered a new narrative in wound physiology: electrical fields act not just on individual cells but on the entire immune epithelial collective, redirecting inflammation, restoring coordination, and re-establishing the tissue’s sense of spatial order (Zhang et al., 2025; Chan et al., 2024; (Zhao et al., 2006, 2022); Gao et al., 2025). Mechanistic studies now link bioelectric signaling, cellular electrophysiology, and immunometabolic regulation to immune defense and tissue repair (Westermann et al., 2025; Xin et al., 2023). Electroceutical treatments influence both innate and adaptive immune responses, extending from macrophages to T and B lymphocytes. Applied electric fields alter membrane capacitance and conductivity, modifying proliferation, migration, and cytokine expression programs in electrically responsive immune populations. Various electrical stimulation paradigms-including direct current, alternating current, and pulsed modalities have been investigated for their immunomodulatory potential, with both waveform characteristics and temporal patterning critically influencing immune responses (Roy Barman and Jhunjhunwala, 2023). While earlier sections described how bioelectric cues operate at molecular, cellular, and microbial levels, these signals ultimately converge at the scale that matters most clinically the entire wound. At this level, electrical modulation does not act uniformly but instead creates a spatial and functional asymmetry that favors host repair programs while simultaneously destabilizing microbial persistence (Figure 4). As explained in Figure 4, whole-of-wound bioelectric modulation establishes a bidirectional signaling landscape in which keratinocytes, fibroblasts, immune cells, and antimicrobial effectors are directionally coordinated toward the wound center, while microbial communities experience electrical stress, EPS disruption, and loss of biofilm integrity. This asymmetric reprogramming enhances host resilience and collective repair while exposing pathogens to physiological vulnerabilities they cannot readily adapt to. The following sections describe how electrical cues influence host responses of wound healing.

### 4.1 Immune modulation

Electrical stimulation enhances phagocytosis, chemotaxis, growth factor release, and macrophage polarization (Bi et al., 2025; Bianconi et al., 2024). These effects support balanced inflammatory resolution and restore coordinated immune responses that often fail in chronic wounds (Wang et al., 2022; Uemura et al., 2023). Electrically modulated macrophages exhibit improved migratory capacity, increased pathogen clearance, and reparative cytokine profiles, contributing to a wound environment that favors tissue regeneration over persistent inflammation (Bi et al., 2025; Bianconi et al., 2024).

#### 4.1.1 A new understanding of how immune cells “read” electricity

Not long ago, the idea that immune cells respond to electrical fields seemed speculative. But recent breakthroughs have changed that picture entirely. By 2025, a team at Trinity College Dublin demonstrated that human macrophages can be directly reprogrammed by precisely controlled electrical stimulation pushing them away from inflammatory phenotypes and into pro-healing modes (O’Rourke et al., 2025). Electrical cues suppressed harmful cytokines and enhanced macrophages’ capacity to support tissue repair. This was the first time such a shift had been shown in human immune cells, marking a turning point in immuno-electroceutical science. This discovery is profound. It suggests the immune system is not only chemically programmable, but electrically programmable.

#### 4.1.2 Rebalancing the “stalled” wound

Electrical stimulation has been repeatedly shown to boost innate immune functions. A 2025 meta-analysis highlighted that electrical cues enhance phagocytosis, chemotaxis, growth-factor release, and macrophage polarization, all essential ingredients of wound repair (Bi et al., 2025). Beyond simply increasing activity, electrical signals appear to influence how immune cells interpret their environment, shifting them toward functional states that favor resolution rather than persistence of inflammation. Experimental studies indicate that electrically exposed macrophages display improved migratory capacity, more efficient pathogen clearance, and altered cytokine profiles consistent with reparative phenotypes. These changes suggest that immune cells are not passive responders to injury but dynamic interpreters of bioelectric cues, integrating electrical information with biochemical and mechanical signals to guide their behavior. These findings tell a compelling story. When immune cells encounter the right electrical environment, they rediscover their original purpose to resolve inflammation and rebuild tissue, rather than lingering in cycles of chronic activation (Bianconi et al., 2024; Wang et al., 2022; Uemura et al., 2023). In chronic wounds, where immune signaling often becomes dysregulated and self-sustaining, this ability to recalibrate immune behavior may represent a critical therapeutic advantage. By restoring directional migration, balanced cytokine production, and coordinated interactions with stromal and epithelial cells, electrical modulation helps shift the wound microenvironment from inflammatory stagnation toward progressive healing.

### 4.2 Epithelial and stromal cell responses: restoration of directional cellular migration

#### 4.2.1 Keratinocytes: electric fields as a navigation system

One of the most visually striking findings of the past 3 years comes from microfluidic electrotaxis platforms. In 2023, researchers showed that keratinocyte sheets migrate three times faster when guided by a uni-directional electric field, even rescuing the impaired motility of diabetic keratinocytes (Shaner et al., 2023). This means keratinocytes do not simply move they sense, interpret, and align themselves to electrical gradients. In a wound environment, where biochemical gradients are noisy and dynamically shifting, electrical cues act as a reliable compass, ensuring the epithelium closes in a coordinated, sheet-like manner. Another study shows how keratinocytes respond to ES through ERK1/2 and p38 MAPK activation, increased proliferation, altered keratin expression, and modulation of cytokines and growth factors, including VEGF, supporting immune recruitment and wound progression (Zhou et al., 2024). Electrically guided collective migration of keratinocyte sheets accelerates re-epithelialization via receptor redistribution and cytoskeletal polarization (Liu et al., 2025). Conductive substrates under ES increase secretion of IL-1α, IL-6, IL-8, GROα, FGF2, and VEGF-A, linking electrical cues to immune recruitment and angiogenesis (Katoh, 2023). Keratinocyte-driven innate defense is also mediated by AMPs, including defensins and LL-37, which integrate antimicrobial activity with immune signaling (Wu et al., 2025).

#### 4.2.2 Fibroblasts, endothelial cells, and the cross-talk of repair

Electrical stimulation activates fibroblasts, keratinocytes, and endothelial cells simultaneously boosting collagen deposition, angiogenesis, and extracellular matrix organization (AbedinDo et al., 2021; Preetam et al., 2024). In effect, electrical fields synchronize the activity of three essential populations: 1. Fibroblasts act as architects of the scaffold. 2. Endothelial cells restore blood supply 3. Keratinocytes seal the surface. These cell types form the triad of regeneration, and electrical cues appear to unify their efforts. This cross-talk among cell types mediated through electrical guidance cues, growth factor gradients, and changes in inflammatory signaling constitutes a coordinated regenerative network rather than a set of isolated cellular responses (Park et al., 2025; Shaner et al., 2023).

Recent advances underscore not only the individual activation of these cells by ES, but also how their interactions are dynamically regulated in space and time to ultimately drive more efficient tissue repair (Zhang et al., 2025). Low-intensity DC stimulation of diabetic human skin fibroblasts reduces IL-6 and IL-8 while increasing growth factor release, shifting the inflammatory-reparative balance of the wound microenvironment (Las Heras et al., 2024). Meta-analyses report demonstrated increased VEGF, FGF, PDGF, EGF, and TGF-*β* under ES, consistent with repair-supportive signaling (Rabbani et al., 2024).

#### 4.2.3 Systems-level modulation of adaptive immune responses

Bioelectric cues also influence adaptive immunity. Charged substrates enhance CD8^+^ T-cell activation, proliferation, and effector differentiation by modulating TCR signaling and metabolic fitness (Song et al., 2025). DC electric fields guide migration of CD4^+^ and CD8^+^ T cells *in vitro*, supporting electrical control of effector positioning (Ende et al., 2024), and low-intensity fields suppress IL-2, Th17 programs, and STAT3 phosphorylation. Charged substrates combined with DC stimulation enhance TCR signaling and early growth response programs. Stromal-immune coupling can also reinforce adaptive responses, as IFNγ-responsive fibroblasts recruit autoreactive CD8^+^ T cells in vitiligo (Xu et al., 2022). Electroporation-based electrical interventions increase antigen uptake and presentation, enhancing CD8^+^ T-cell responses and Langerhans cell activation. Pulsed electric fields further promote adaptive immune infiltration and activation in tissue environments (Salameh et al., 2024).

### 4.3 The coordination of the whole tissue: how electrochemical signals promote coordinated tissue responses

#### 4.3.1 Restoring communication in disrupted tissue

Cutaneous wounds disrupt not just individual cells but the communication networks that allow tissues to function as coordinated systems. Injury interrupts calcium signaling waves, mechanical alignment, and gap-junction continuity, fragmenting the collective behavior that normally guides repair. As a result, cells near the wound edge may receive inconsistent signals, contributing to disorganized migration, prolonged inflammation, and impaired tissue reconstruction. Modern bioelectronic platforms show that applied electrical fields can help restore these lost communication circuits, promoting synchronized cellular responses and re-establishing coherent collective behavior (Jiang et al., 2025; Karunasagara et al., 2025).

Electrical cues appear to facilitate intercellular coordination by stabilizing membrane potentials, enhancing ion flux continuity, and supporting gap-junction mediated signaling across epithelial and stromal layers. This promotes more uniform calcium oscillations, directional migration, and coordinated activation of repair programs across the wound field. Rather than acting on isolated cells, electrical modulation helps tissues regain system-level organization, allowing multiple cell populations to respond in a temporally aligned and spatially coherent manner. Restoring this communication framework is particularly important in chronic wounds, where disrupted signaling networks contribute to stalled healing trajectories.

#### 4.3.2 Real-time sensing + electrical correction

Last year saw the emergence of smart cutaneous bioelectronic interfaces, capable of simultaneously monitoring pH, temperature, oxygenation, and infection markers while delivering corrective electrical stimulation. These systems allow the host to receive immediate, tailored electrical cues that stabilize inflammation and accelerate reconstruction (Jiang et al., 2025). These transform wound care from a reactive process to a closed-loop dialogue. These systems enable real-time physiological monitoring with responsive stimulation, and the stimulation responds. Furthermore, non-contact electrical stimulation modalities, such as capacitive coupling, have been demonstrated to direct macrophage polarization toward the pro-healing M2 phenotype, characterized by decreased CCR7 and increased CD206 expression, thereby enhancing immune regulation during wound repair (Xu et al., 2022). Emerging wearable electroceutical platforms that integrate electrical stimulation with real-time sensing of pH and infection biomarkers facilitate dynamic modulation of immune responses, attenuating inflammation and promoting tissue regeneration (Vo and Trinh, 2025).

### 4.4 A new perspective: electrical modulation of coordinated tissue repair

The emerging literature paints a powerful picture. Electrical cues are not merely external inputsthey represent an important component of tissue coordination. Immune cells can shift from inflammatory to reparative identities (Mutah et al., 2025; Zhao et al., 2025). Keratinocytes regain directional coordination. Fibroblasts and endothelial cells orchestrate ECM rebuilding and angiogenesis. Smart electroceutical interfaces maintain real-time communication within the wound (Bi et al., 2025). Electricity does not fight biology – it reminds biology how to heal. This is why modern research increasingly sees electrochemical modulation as a system-level therapy, capable of rewriting wound trajectories that have stalled for months or even years (Gao et al., 2025).

## 5 Clinical applications: where electrical modulation meets the realities of human wounds

**Figure 5:**
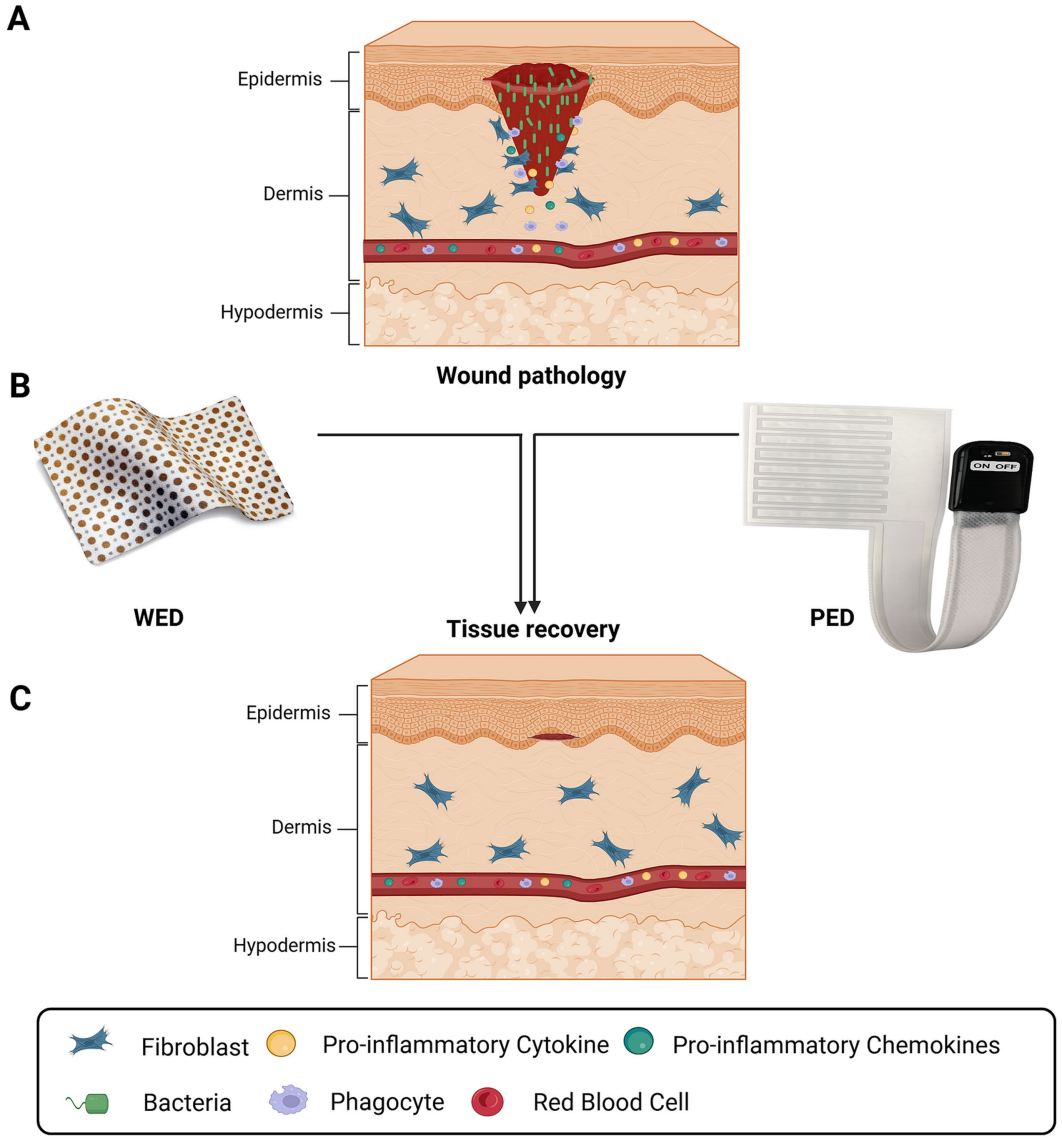
Clinical electroceutical platforms for wound repair: **(A)** Cutaneous injury disrupts epidermal integrity, enabling bacterial colonization, inflammatory cytokine release, and immune cell infiltration within the wound bed. **(B)** Wireless electroceutical dressings (WED) and powered electroceutical devices (PED) are applied to the wound surface to deliver controlled electrical stimulation directly to injured tissue. **(C)** Electroceutical treatment suppresses infection-driven inflammation, promotes fibroblast activity, and restores epithelial and dermal architecture, resulting in accelerated wound closure and tissue regeneration. Created in BioRender. Bhasme, P. (2026). https://BioRender.com/fb7rmrf.

Clinically deployed electroceutical platforms leverage these principles to reshape wound bioelectric and inflammatory environments, supporting coordinated tissue recovery across diverse wound contexts (Figure 5). Clinical evidence for electrical intervention in wound healing continues to expand in clinical settings. There, in the unpredictable terrain of diabetic ulcers, burns, surgical incisions, and chronically infected wounds, electrical and electrochemical cues have begun to reshape therapeutic possibility (Evans and Sen, 2023). Over the last 3–5 years, clinical science has accelerated rapidly driven by new devices, smarter biomaterials, and a deeper understanding of wound bioelectric physiology (Bi et al., 2025; Mishra et al., 2024). The mechanistic principles described above have enabled translation of electroceutical strategies into clinical and preclinical wound-care applications.

### 5.1 Chronic and infected wounds: restoration of impaired healing environments

Chronic wound diabetic foot ulcers, venous leg ulcers, pressure injuries are not merely delayed injuries; they are electrically derailed ecosystems. Their endogenous fields are weakened, their epithelial edges unresponsive, and their immune systems trapped in sterile inflammation. Chronic wounds such as diabetic foot ulcers, venous leg ulcers, and pressure injuries display persistent defects that limit responsiveness to standard care, including disrupted endogenous electrical cues, biofilm colonization, and prolonged inflammation (Shen et al., 2025; Singh et al., 2025). Electroceutical therapies act at the wound interface to reinforce weakened electrical gradients that guide epithelial organization while simultaneously destabilizing biofilm structure and metabolic coordination (Evans and Sen, 2023). Key clinical applications of electroceutical wound therapies across wound types and device platforms are summarized in Table 2. The table integrates evidence from preclinical and clinical studies to illustrate how electrical modulation is deployed in chronic wounds, burns, and surgical sites, highlighting therapeutic objectives, device modalities, and observed outcomes. By consolidating these applications, the table clarifies how mechanistic bioelectric principles translate into practical wound-care strategies across diverse clinical contexts.

**Table 2:** Wound-type specific clinical deployment of electroceuticals in infection control and tissue repair.

Wound type	Dominant pathophysiology	Key clinical electroceutical targets	Clinical/functional outcome	References
Diabetic foot ulcers (DFU)	Neuropathy + ischemia + impaired repair signaling	Adjunct electrical stimulation to accelerate closure	Faster healing vs. standard care across RCTs (summary evidence)	Lan et al. (2024)
DFU (home-based care)	Limited clinic access; poor perfusion	Wearable/home E-stim adjunct	Feasibility + improved healing metrics in chronic DFU	Zulbaran-Rojas et al. (2023)
DFU (evidence synthesis)	Chronic inflammation and stalled granulation	Electrostimulation as adjunct to usual wound care	Overall benefit signal without increased adverse events (evidence report)	Huang et al. (2023)
Burn wounds (biofilm infection risk)	Rapid colonization + biofilm-driven delayed healing	Wireless electroceutical dressing vs. SOC	Reduced biofilm burden/improved infection control endpoints	Chan et al. (2024)
Burn wounds (therapy comparison)	Variable depth + heterogeneous wound bed	Microcurrent/wearable stimulation modalities	Shorter healing time signal in clinical cohort	Koh et al. (2025)
Burn wounds (overall evidence)	Infection risk + scarring	ES adjunct across adult burn studies	Evidence synthesis of benefit/limitations	Edwick et al. (2025)
Surgical incisions (next-gen)	Dehiscence + local infection risk	Self-powered mechanoelectric suture	Accelerated healing + antibacterial effect in translational model	Xu Y. et al. (2025)
Surgical wounds (bioelectronic suture)	Need for local therapy without devices	Body-coupled electrotherapy suture	Local ES supports migration/angiogenesis + infection prevention	Sun et al. (2024)
Post-operative (pain + recovery)	Pain control impacts mobility/healing	Noninvasive high-frequency stimulation	Reduced pain/opioid outcomes in surgical recovery context	Grasch et al. (2023)
Pressure injuries (evidence)	Ischemia reperfusion + repeated load	Electrical stimulation adjunct	Systematic review of clinical trials	Szołtys-Brzezowska et al. (2023)
Pressure injuries (ongoing RCT)	Early-stage sacral/ischial breakdown	Intermittent ES + routine care	Multicenter RCT evaluating ulcer reduction vs. routine care	Donaldson et al. (2024)
Venous leg ulcers (chronic)	Venous hypertension + inflammation	ES adjunct to compression/SoC	Evidence summary: potential improved healing	Huang et al. (2023)
Chronic infected wounds (materials)	Mixed infection + oxidative stress	Conductive polymer bioelectronics	Antibacterial/anti-inflammatory + regeneration support	Jiang et al. (2025)
Polymicrobial chronic wounds	Mixed species biofilms	Anti-biofilm therapy landscape incl. Electroceuticals	Updated clinical significance + anti-biofilm strategies	Cavallo et al. (2024)
Chronic wounds (technology review)	Multiscale impairment	Electrical stimulation modalities	Recent review of mechanisms + translation barriers	Preetam et al. (2024)
Chronic wounds (practice guidance)	Variable etiology	ES as adjunct across wound types	Broad synthesis of advances and clinical framing	Bi et al. (2025)
Health economics (chronic wounds)	Cost burden + recurrence	ES + SoC vs. SoC	Cost-effectiveness synthesis/modeling	Smith et al. (2025)

#### 5.1.1 Re-establishing bioelectric order in diabetic ulcers

Recent analyses of electrical stimulation in chronic wounds reveal consistent benefits: faster closure rates, improved granulation, and reduced infection. Meta-analyses published in 2023–2024 consolidate evidence across dozens of trials showing that electrical stimulation enhances early-stage healing processes and restores directional cell migration in diabetic ulcers, where bioelectrical signaling is normally impaired. Electrical modulation shapes the inflammatory milieu by limiting excessive protease activity and preserving extracellular matrix components involved in keratinocyte attachment and migration (Shoko and Sigauke, 2023). In metabolically compromised wounds where vascular insufficiency and oxidative stress attenuate repair signaling, applied electrical cues can partially compensate for reduced endogenous bioelectric activity and support coordination of re-epithelialization without directly forcing proliferative programs (Tran et al., 2023).

#### 5.1.2 Wearable microcurrent platforms for everyday care

Modern wearable electroceutical devices-including flexible microcurrent patches, triboelectric generators, and piezo-nanogenerators allow patients to receive continuous low-intensity stimulation throughout daily activity (Barman et al., 2023). These devices require no external power source and are designed for home use, directly addressing longstanding barriers in chronic-wound management such as limited clinic access and poor adherence (Fang et al., 2025). These innovations empower patients with chronic wounds to carry their therapy with them quiet, continuous, and body-integrated. Wearable formats address barriers associated with long-term wound management by integrating therapy directly into routine care and enabling continuous modulation without active patient operation or external power sources (Mishra et al., 2024). Passive delivery reduces dependence on adherence to dosing schedules and minimizes disruption to daily activity. Conformable materials improve durability across anatomical sites subject to motion or pressure, while patient-centric features may reduce dressing change frequency and clinical visits in populations requiring extended wound care (Chan et al., 2024; Moradifar et al., 2025).

### 5.2 Burn wounds: electrical fields as early firebreaks against infection

Burns are uniquely vulnerable to microbial invasion they present a warm, nutrient-rich environment with exposed tissues and compromised vasculature. The first hours after injury are critical, and electrical interventions provide a powerful early advantage. Burn wounds and surgical site injuries are particularly susceptible to microbial colonization due to exposed tissue surfaces, immune dysregulation, and increased nutrient availability. Electroceuticals operate directly at the wound surface, limiting early microbial attachment and constraining biofilm establishment while preserving viable tissue margins (Chan et al., 2024). In contrast to topical antimicrobials, electrical modulation does not rely on diffusion through necrotic tissue, enabling more uniform activity across heterogeneous wound beds.

#### 5.2.1 Accelerating epithelial recovery in burns

Clinical studies using microcurrent and controlled electric-field devices demonstrate significantly faster re-epithelialization in burn wounds (Edwick et al., 2025). Improved collagen organization and more orderly tissue remodeling have been noted, highlighting the ability of electrical cues to restore structural symmetry in wounds where architecture has been severely disrupted. These effects align with the concept that electrical modulation stabilizes wound edges and supports coordinated tissue repair at the interface of inflammation control and barrier restoration, particularly during early phases when burn margins remain viable and responsive to guidance cues (Donaldson et al., 2024).

#### 5.2.2 Preventing early biofilm formation

Burn wounds often become infected before they even finish cooling. Electrical stimulation disrupts early biofilm attachment and maturation, effectively buying time for the immune system and topical therapies to act. These findings echo laboratory data showing microbial membrane depolarization and weakened quorum behavior under mild electrical influence. By limiting early microbial attachment at the wound surface and constraining biofilm establishment, electroceuticals can reduce microbial thresholds that otherwise necessitate escalation to systemic therapy, particularly when burn microenvironments promote rapid colonization (Chan et al., 2024).

### 5.3 Surgical wounds: bioelectronic stabilization of incisional healing and complication prevention

Surgical incisions generally follow a more predictable healing trajectory than chronic wounds; however, postoperative sites remain vulnerable to infection, dehiscence, and delayed closure, particularly in patients with impaired perfusion, metabolic disease, obesity, or immunosuppression. Disruption of local tissue organization, inflammatory imbalance, and microbial contamination during the early postoperative window can destabilize the coordinated repair processes required for reliable closure. Electrical and electrochemical modulation offers a strategy to reinforce these early healing dynamics by influencing both host tissue organization and microbial activity at the incision interface (Evans and Sen, 2023; Mishra et al., 2024).

Recent advances in cutaneous bioelectronic interfaces demonstrate that localized electrical stimulation applied along incision lines supports more ordered collagen deposition, reduces inflammatory dysregulation, and improves structural coherence during wound maturation. Electrically active dressings appear to stabilize wound edges by promoting coordinated fibroblast activity and extracellular matrix alignment, factors closely associated with reduced risk of dehiscence in high-risk surgical populations (Kramer et al., 2023). Electrical modulation of fibroblast behavior and matrix organization has been linked to improved reparative signaling and angiogenic support, mechanisms that contribute to stabilization of surgical wound architecture (Preetam et al., 2024; Park et al., 2025). Integration of sensing capabilities within these platforms further enables early detection of infection-related physiological changes, such as alterations in pH, temperature, or impedance, allowing timely therapeutic adjustment (Wang et al., 2025). Prophylactic application immediately following incision closure has also been explored as a means of modifying early wound microenvironments that predispose to infection, particularly in patients with vascular compromise or systemic comorbidities (Daryapeyma et al., 2025).

Preclinical surgical models further support the translational potential of this approach. Studies in large-animal systems, including porcine incision models, demonstrate that wireless electroceutical dressings applied over surgical sites improve vascular perfusion, reduce bacterial contamination, and promote more rapid tissue integration (El Masry et al., 2023). These platforms employ low-intensity direct current fields to simultaneously influence microbial energetics and host repair coordination without inducing tissue damage, consistent with observations that mild electrical cues can destabilize microbial electrochemical homeostasis while preserving host tissue viability (Lee et al., 2024; Ungureanu et al., 2025).

Increasingly, electroceutical systems incorporate embedded sensing technologies capable of monitoring wound parameters such as pH, temperature, moisture, electrical impedance, and metabolic markers, providing continuous insight into wound status and therapeutic response. Sensor-enabled platforms allow stimulation intensity to be dynamically modulated in response to evolving wound conditions rather than applied as a fixed regimen, limiting unnecessary tissue stress while maintaining antimicrobial and regenerative effects (Kramer et al., 2023; Wang et al., 2025). Integration with remote monitoring infrastructure further supports clinician oversight without frequent in-person evaluation, enabling individualized care trajectories across outpatient and home-based settings (Rahman et al., 2025; Liu et al., 2025).

### 5.4 Applications in high-risk and systemically compromised wound environments

Delayed or impaired wound healing frequently arises not solely from local tissue disruption but from systemic factors such as vascular insufficiency, immune dysregulation, metabolic disease, or chronic inflammation that compromise the body’s capacity to coordinate repair. In these contexts, interventions that modulate the wound microenvironment without imposing excessive biological stress are particularly relevant. Electrical and electrochemical therapies have shown emerging potential in such high-risk settings by influencing inflammatory balance, tissue organization, and microbial burden simultaneously (Evans and Sen, 2023; Bi et al., 2025). Because bioelectric cues operate upstream of conventional molecular signaling pathways, they offer an approach capable of restoring coordination across multiple biological systems rather than targeting isolated pathways (Balasubramanian et al., 2024; Mishra et al., 2024).

Evidence supporting this approach is beginning to emerge in populations characterized by immune compromise or impaired regenerative capacity. Experimental studies demonstrate that controlled electrical stimulation can shift human macrophages toward pro-regenerative and anti-inflammatory phenotypes, suggesting a mechanism by which bioelectrical cues may help rebalance dysregulated immune responses in vulnerable patient groups (O’Rourke et al., 2025; Bianconi et al., 2024). Rather than broadly suppressing inflammation, electrical modulation appears capable of guiding immune activity toward reparative trajectories while preserving host defense capacity. Electrical stimulation has also been associated with improved growth-factor release, angiogenic signaling, and cellular coordination in metabolically compromised wound environments, supporting recovery in conditions where endogenous repair signaling is attenuated (Preetam et al., 2024; Gao et al., 2025). Consistent with this concept, prophylactic or early application of electroceutical interventions has been explored in patients with immunosuppression or vascular disease to modify early wound conditions that predispose to infection and delayed closure (Daryapeyma et al., 2025).

#### 5.4.1 Mixed-etiology chronic wounds

Wearable and adaptive systems allow dynamic adjustment based on real-time wound status pH, temperature, oxygenation helping clinicians respond to fluctuating challenges such as intermittent ischemia or polymicrobial infections. Clinical response variability is influenced by wound etiology, timing of intervention, and integration with standard care practices. Factors including wound moisture, microbial burden, tissue perfusion, and exudate composition shape electrical activity and therapeutic effects within the wound environment, underscoring the importance of contextual deployment rather than uniform application (Huo et al., 2025). Electroceutical platforms incorporating biosensing and responsive stimulation architectures enable modulation of electrical output according to local physiological conditions, which may influence epithelial migration, angiogenesis, and immune regulation across heterogeneous wound etiologies (Kramer et al., 2023; Mishra et al., 2024; Wang et al., 2025). In mixed-etiology wounds where ischemic, inflammatory, and infectious components coexist, modulation of local bioelectric conditions has been associated with improvements in vascular perfusion, cellular coordination, and microbial control within compromised tissue environments (Evans and Sen, 2023; Bi et al., 2025; Tran et al., 2023). Electrically active dressings and conductive biomaterials have also been reported to influence extracellular matrix organization and angiogenic signaling in complex wound beds characterized by impaired perfusion and metabolic stress (Preetam et al., 2024; Park et al., 2025).

#### 5.4.2 Elderly and mobility-limited populations

Because many electroceutical systems can be worn continuously, they reduce the need for frequent dressing changes, lowering caregiver burden and improving patient autonomy. Patient-centric features may reduce dressing change frequency and clinical visits, supporting sustained therapeutic strategies in populations requiring extended wound care (Moradifar et al., 2025). Continuous low-intensity stimulation delivered through flexible or self-powered platforms has been investigated in contexts where impaired perfusion, reduced cellular responsiveness, and delayed immune coordination contribute to prolonged healing trajectories, including aging-associated wound environments (Mishra et al., 2024; Gao et al., 2025). Wearable electroceutical interfaces that maintain stable electrical microenvironments over extended periods have been associated with modulation of inflammatory signaling, cellular migration, and angiogenic responses, while integrated sensing capabilities allow monitoring of infection-related physiological parameters without frequent clinical intervention (Kramer et al., 2023; Wang et al., 2025; Rahman et al., 2025). Conformable and lightweight device architectures have also been reported to improve usability across anatomical sites subject to pressure or limited mobility, supporting adherence in patients with restricted ambulation or caregiver dependence (Chan et al., 2024; Park et al., 2025).

### 5.5 The rise of closed-loop, AI-enhanced electroceutical care

The most exciting clinical frontier is the convergence of real-time sensing, adaptive electrical stimulation, and AI-driven decision engines. Cutaneous bioelectronic interfaces already monitor pH, temperature, oxygen tension, and infection biomarkers adjusting electrical output to match healing phase and microbial threat (Vo and Trinh, 2025). This dynamic approach reduces overtreatment, prevents tissue fatigue, and accelerates trajectory toward closure (Turki and Alrafiah, 2025). Sensor-enabled electroceutical systems provide continuous insight into wound status, enabling detection of non-visible clinical deterioration and allowing output to be modulated in response to evolving wound conditions rather than applied as a fixed regimen (Kramer et al., 2023; Wang et al., 2025). Adaptive control limits unnecessary tissue stress while maintaining antimicrobial and regenerative effects, and remote monitoring infrastructure supports clinician oversight without frequent in-person evaluation, enabling individualized care trajectories.

### 5.6 Why electrical interventions may thrive where drug therapies fail

Across these diverse wound types, one theme repeats. Electrical modulation may offer advantages in contexts where purely biochemical therapies are insufficient. Why? Because these interventions do not rely on single molecular interactions. They reshape membrane potential, cell migration cues, microbial energetics, immune-cell programming, redox environment, and tissue-level coordination. They act on physical principles underlying wound biology that microbes may have limited ability to adapt around and host tissues are organized to interpret (Qin and Tape, 2024). Electroceuticals align reduce reliance on systemic antibiotics by lowering microbial and inflammatory thresholds that otherwise necessitate prolonged antimicrobial therapy, particularly by weakening biofilm cohesion and disrupting membrane energetics to support local immune clearance (Bhuiyan, 2025). Because this modality operates largely independently of classical resistance mechanisms and does not rely on target-specific inhibition, it can be integrated alongside antimicrobial therapy without substantially amplifying selective pressures that contribute to resistance emergence (Magisetty and Park, 2022). Translation is shaped by regulatory pathways requiring demonstration of consistent electrical output under variable wound conditions, including differences in moisture, exudate composition, and tissue contact, alongside safety assessment for prolonged exposure and biological impact (Kramer et al., 2023). Additional translational challenges include defining stimulation parameters appropriate for diverse wound etiologies, establishing outcome measures beyond closure rates, and characterizing longer-term effects on commensal microbiota (Weigelt et al., 2022). Ongoing studies continue to refine optimal use scenarios, parameter ranges, and combinations with adjunctive therapies in wounds complicated by infection, ischemia, or metabolic dysfunction (Fu et al., 2026).

### 5.7 Clinical scope and implementation considerations

Clinical use demonstrates variability in response influenced by wound etiology, timing of intervention, and integration with standard care practices. Factors including moisture, microbial burden, perfusion, and exudate composition shape electrical activity and therapeutic effects within the wound environment (Huo et al., 2025). Variations in tissue conductivity, electrode–tissue interface characteristics, and wound geometry can influence current distribution and field strength, contributing to heterogeneity in therapeutic outcomes across patients and wound types (Kramer et al., 2023; Mishra et al., 2024). Differences in inflammatory burden, vascular integrity, and metabolic status further modulate cellular responsiveness to electrical cues, particularly in chronic wounds associated with systemic disease or impaired perfusion (Evans and Sen, 2023; Bi et al., 2025).

Integration with routine clinical workflows remains an important determinant of successful implementation. Device usability, dressing compatibility, treatment duration, and patient adherence influence real-world performance, particularly in outpatient and home-care settings (Chan et al., 2024; Moradifar et al., 2025). Sensor-enabled electroceutical systems capable of monitoring physiological parameters such as pH, temperature, moisture, and impedance provide additional clinical information that may support treatment adjustment and early detection of complications (Wang et al., 2025; Kramer et al., 2023). Clinician familiarity and workflow integration remain central to success, and emerging technologies and data-driven approaches are expected to further refine how electroceutical interventions are deployed and personalized (Fu et al., 2026).

## 6 Future direction

**Figure 6:**
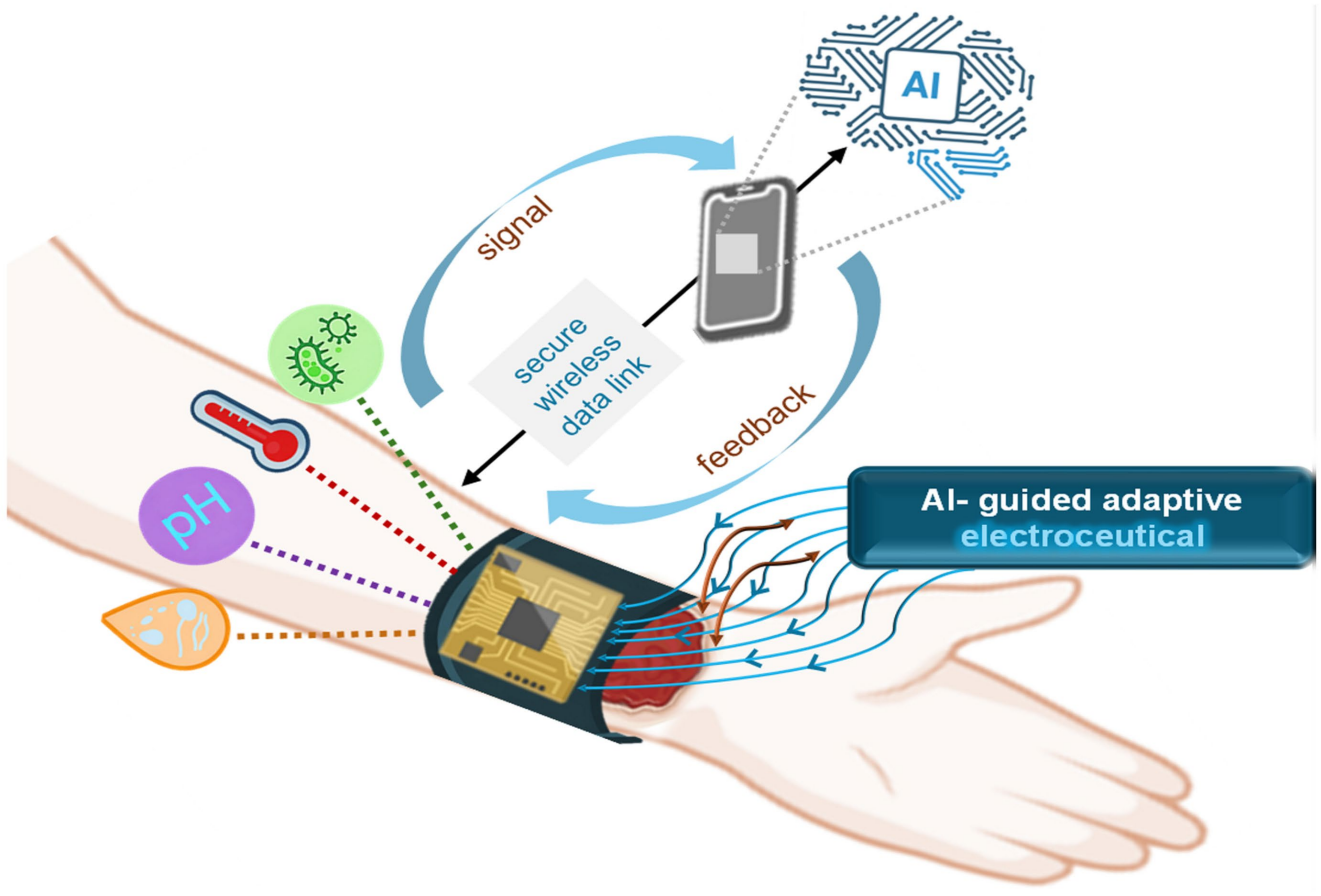
Advance AI-integrated electroceutical system. A closed-loop smart bandage integrates sensors for pH, temperature, and infection-associated signals with wireless data transmission to an AI-driven platform. Machine-learning–guided analysis dynamically adjusts electrical stimulation in real time, enabling adaptive and personalized electroceutical wound therapy. Created in BioRender. Bhasme, P. (2026). https://BioRender.com/fb7rmrf.

Despite rapid progress, electroceutical wound therapy remains at the edge of its full potential. Recent reviews and systematic analyses highlight extraordinary promise accelerated healing, microbial suppression, improved angiogenesis but they also expose foundational gaps in understanding that currently limit translation. Solving these unanswered questions would enable electroceuticals to shift from an adjunctive modality into a central pillar of wound management (Khalpey et al., 2025; Kim et al., 2026). Building on these advances, emerging electroceutical platforms are increasingly designed as closed-loop systems that integrate real-time sensing, wireless communication, and artificial intelligence to dynamically adapt electrical therapy to the evolving wound environment (Figure 6).

### 6.1 What are the optimal dosing parameters for electrical stimulation in human wounds?

Across clinical and preclinical studies, electrical stimulation has been shown to manage biofilm infection, enhance keratinocyte and fibroblast activity, accelerate angiogenesis, and improve closure rates (Preetam et al., 2024; Peng et al., 2025; Jonik et al., 2025). Yet no unified stimulation protocol exists waveforms, intensities, durations, and frequencies vary dramatically between studies. A 2024 meta-analysis emphasized that inconsistency in experimental design prevents meaningful comparison or reproducibility and remains one of the largest barriers to clinical adoption (Rabbani et al., 2024). Similarly, a 2025 review called for mechanistically informed optimization of endogenous and exogenous electrical cues, noting that many studies still rely on trial-and-error parameter selection rather than physiologically grounded design (Bi et al., 2025). The open question: What are the mechanistically optimal electrical “doses” for different wound types, phases, and patient populations? A breakthrough here would allow the field to shift from empirical stimulation to precision electrotherapy.

### 6.2 How do electrical cues interact with complex immune environments in chronic wounds?

The immune response is one of the most promising yet least understood domains of electroceutical therapy. A landmark 2025 study demonstrated that electrical stimulation can directly reprogram human macrophages, suppress inflammatory phenotypes and enhancing reparative behaviors (O’Rourke et al., 2025). But this raises deeper questions. How do electrical fields influence immune heterogeneity in polymicrobial infections? Can directed stimulation prevent immune exhaustion in chronic ulcers? How do T-cell, neutrophil, and dendritic cell electro sensitivities differ? Given that chronic wounds are fundamentally immunological failures, answering these questions may unlock electro-immunotherapy for wounds.

### 6.3 What electrochemical signatures predict wound deterioration or recovery?

Electroceutical technologies are transitioning from static stimulators into adaptive bioelectronic systems capable of sensing, interpreting, and modulating the wound microenvironment in real time (Bi et al., 2025). Smart bioelectronic interfaces introduced in 2025 can integrate electrical stimulation with continuous physiological monitoring, allowing therapy to be dynamically adjusted based on biomarkers such as inflammation, metabolic status, temperature, oxygenation, and infection markers in real time (Rizzo et al., 2025). These systems promise closed-loop treatment but expose a profound unknown: which electrical and biochemical signals reliably indicate trajectory toward healing versus deterioration? Without validated biomarkers, closed-loop systems cannot reach full autonomy. Electroactive nanofibrous scaffolds, conductive hydrogels, and microcurrent dressings provide spatially uniform electrical cues that guide keratinocyte and fibroblast migration while supporting endothelial proliferation and extracellular matrix assembly (Fang et al., 2025).

These materials also serve as platforms for integrating antimicrobial and immunomodulatory functions, enabling electroceuticals to simultaneously control infection and regeneration. Emerging electroceutical materials increasingly couple bioelectric stimulation with immune and antimicrobial mechanisms (Turki and Alrafiah, 2025; Liu et al., 2025).

Next,-generation intelligent electrospun nanomaterials integrate sensing, actuation, and signal transmission into a single platform (Uyanga et al., 2025). These materials detect pH, temperature, glucose, humidity, and bacterial metabolites and convert them into optical or electrical outputs that can trigger therapeutic responses, transforming electroceuticals into active participants in wound surveillance and control (Sharma et al., 2025). By incorporating real-time measurements of oxygenation, temperature, and impedance, these systems infer wound status and adjust stimulation to optimize epithelialization, immune activity, and vascularization (Shan et al., 2025). AI-enabled conductive hydrogel dressings expand this personalization by combining soft, hydrated matrices with conductive fillers and embedded sensors that support epithelial migration while continuously monitoring wound conditions (Zhao et al., 2025). Machine-learning models integrate multisignal data streams to detect early signs of infection, ischemia, or stalled healing and adjust therapy before clinical deterioration occurs (Almeida et al., 2025; She et al., 2025). Image-guided bioelectronic systems provide complementary feedback. Platforms such as a-Heal use automated wound imaging and AI-based interpretation to determine wound stage and prescribe optimal electrical or pharmacologic interventions, updating therapy as healing progresses (Liu et al., 2025). Deep-learning ensembles trained on longitudinal wound images generate severity scores and healing-rate predictions that inform real-time adjustment of electroceutical therapy (Thanha et al., 2025).

### 6.4 How do electroceuticals reshape biofilms at species, strain, and community levels?

Wearable electroceutical devices clearly disrupt microbial viability, but mechanisms remain poorly defined, and device designs vary widely. How do specific pathogens (e.g., *P. aeruginosa, S. aureus*) respond to different field patterns? Do electrical cues alter interspecies interactions within polymicrobial biofilms? What redox or membrane-potential thresholds cause irreversible collapse of microbial communities? Defining these principles could yield biofilm-specific electroceuticals for infection control.

Bioelectronic smart bandages integrate continuous sensing with machine-learning-based decision engines that interpret physiological signals and dynamically regulate electrical stimulation (Turki and Alrafiah, 2025). The integration of electroceuticals with AI-driven feedback is particularly impactful in infected and chronic wounds, where biofilms, immune dysfunction, and metabolic stress create unstable and treatment-resistant microenvironments (Wu et al., 2025). In these settings, electroceutical platforms dynamically shift between antimicrobial and regenerative modes by interpreting real-time wound signals such as pH, temperature, moisture, and bacterial metabolites, enabling therapy to adapt to evolving biological conditions (Zhu et al., 2025).

Closed-loop systems that combine electrical stimulation with controlled antimicrobial release suppress biofilm formation while preserving epithelial and stromal cell function, enabling faster and more coordinated healing than static treatments (Mack et al., 2025). These systems exploit differences in the electrical sensitivity of microbes and host tissues, destabilizing microbial electrochemical activity while preserving host cell migration and reparative signaling. In high-risk clinical scenarios, electroceutically active dressings reduce post-surgical infection rates by creating wound environments inhospitable to bacterial colonization and biofilm persistence, including pathogens such as *Pseudomonas aeruginosa* (Khalpey et al., 2025). Preclinical infected-wound models further show that electrically active dressings suppress bacterial burden while enhancing angiogenesis, collagen deposition, and immune-cell recruitment. Continuous biosensing of pH and inflammatory markers enables early detection of microbial overgrowth and dynamic adjustment of therapy, reducing reliance on systemic antibiotics (Chen et al., 2024).

### 6.5 What device architectures maximize efficacy while ensuring usability?

Piezoelectric dressings, triboelectric generators, soft bioelectronics, and microcurrent wearables offer extraordinary possibilities, but recent materials review stressed unresolved challenges including limited portability, insufficient power durability, and incomplete understanding of long-term tissue-device interactions (Dai et al., 2024). The key unknown is: What combination of material, geometry, and electrochemical output best harmonizes with wound physiology?

Piezoelectric dressings convert mechanical energy from body movement or physiological motion into electrical stimulation, enabling self-powered electrical output without batteries. These constructs often use engineered polymers or composites tailored for sustained bioelectric generation and can promote cell proliferation and migration at wound sites (Zhao et al., 2025). Moreover, piezoelectric hydrogels and electrospun scaffolds integrate hydrogel matrices or nanofiber networks with piezoelectric phases to combine soft, tissue-like mechanics with electromechanical transduction. Such as piezoelectric electrospun membranes incorporating gallium-doped bioactive glass generate reactive oxygen species under mechanical activation, suppress bacterial growth, and bias macrophages toward reparative M2 phenotypes, reshaping the wound microenvironment toward healing (Liu et al., 2025). Their flexibility, moisture maintenance, and continuous electrical output better mimic extracellular matrix behavior while reducing patient discomfort. Multi-layer Electret Patches use stacked dielectric layers that sustain a direct current electric field without external electronics. It has been shown that by adjusting layer composition and geometry, these devices can tune field strength to bias fibroblast and other cell behaviors, reducing fibrosis and supporting regenerative outcomes (Liu et al., 2025; Bi et al., 2025).

### 6.6 How can electroceuticals be integrated into standardized clinical workflows?

A systematic review of wearable electroceuticals revealed no standardized clinical pathways, no agreement on patient selection, and limited comparative trials. To become transformative, electroceuticals must evolve from experimental add-ons to fully integrated clinical tools with evidence-based guidelines (Mutah et al., 2025). The convergence of electroceutical materials, biosensing, and AI-driven control establishes a framework for precision wound care in which therapy is continuously adapted to the patient’s biological state (Leng and Sun, 2025). Translation into routine clinical use will require validation of closed-loop performance, including demonstration that AI-guided modulation improves healing outcomes relative to static stimulation or conventional dressings (She et al., 2025).

Regulatory and manufacturing considerations will also shape deployment. AI-integrated electroceuticals must meet evolving regulatory frameworks for combination products that integrate biosensors, therapeutic devices, and software as medical device components. Scalable fabrication of soft, stretchable, and biocompatible electronics is needed to support prolonged wear, repeated sterilization, and consistent performance across patient populations (Xu J. et al., 2025).

Integration with telemedicine and digital health infrastructure enables remote wound monitoring, algorithm-guided therapy updates, and early clinical intervention without frequent in-person visits, improving continuity of care (Rahman et al., 2025). By coupling continuous biosensing with AI-driven control, electroceutical systems provide a scalable model for personalized, data-driven wound management that adapts to infection status, inflammatory burden, and regenerative progress in real time (Liu et al., 2025).

## 7 Conclusion

**Figure 7:**
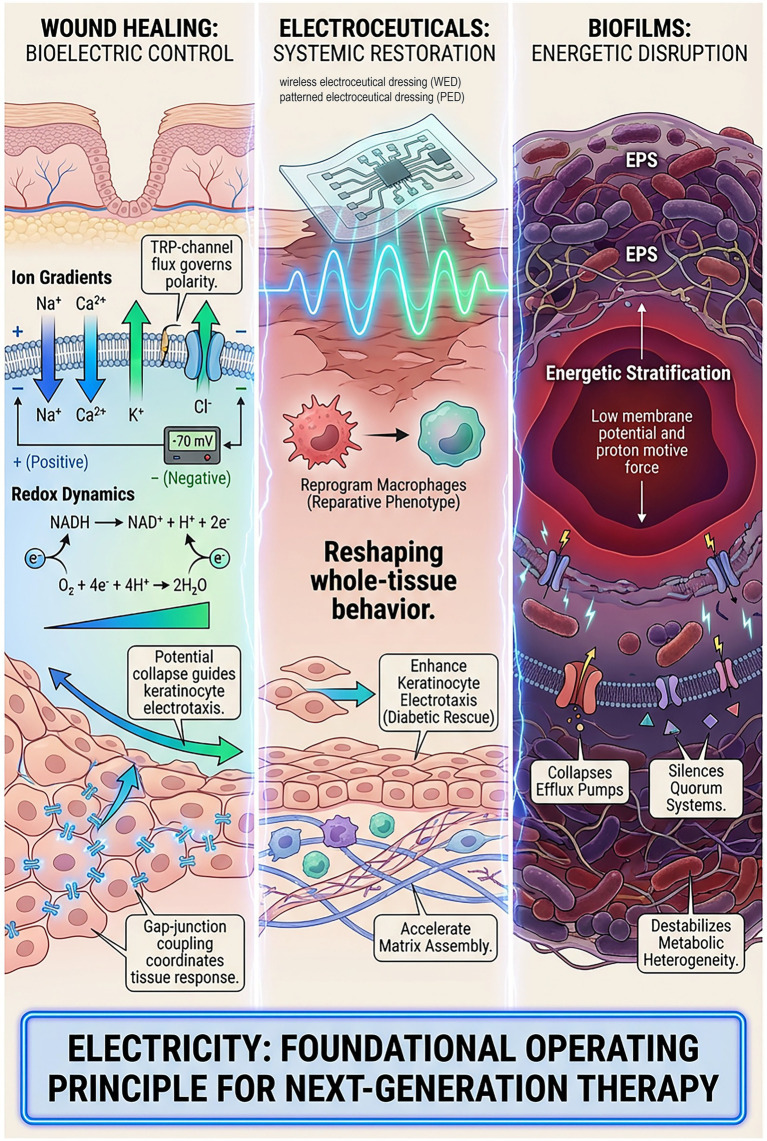
Electricity as a foundational operating principle for next generation therapy. Electrical cues simultaneously restore host tissue organization and destabilize microbial resilience. Depicts three synergistic roles of electrical signaling in advanced wound care: (left) bioelectric control of cellular coordination and redox balance during wound healing; (center) electroceutical modulation of tissue behavior, immune programming, and extracellular matrix assembly; and (right) energetic disruption of microbial biofilms through collapse of membrane potential, efflux systems, and metabolic coordination. Illustration generated employing Google Nano Pro.

The next great leap in electroceutical wound care will come not from stronger devices but from deeper understanding. By answering these core unknowns, the field could move from promising innovation to paradigm-shifting therapy capable of reversing chronicity, preventing infection, and restoring the body’s innate healing intelligence. The capacity of electroceuticals to selectively disadvantage microbes while preserving host cell function creates a unique therapeutic asymmetry, particularly relevant in chronic, polymicrobial, and antibiotic-refractory infections. Clinical translation of electroceuticals has progressed rapidly, which is driven by advances in conductive biomaterials, wireless and self-powered devices, and dressing-integrated platforms. These systems have demonstrated efficiency in variable conditions. Chronic wounds, infected surgical sites, burns, and implant-associated infections have been studied for translational relevance. Emerging smart and adaptive electroceutical platforms have further extended the potential through real-time sensing and closed-loop controls which enables therapy to dynamically respond to evolving biological conditions (Figure 7).

Future integrations of electroceuticals with artificial intelligence, bioresponsive materials, and personalized frameworks aims to redefine infection management and regenerative therapy. Maturing regulatory pathways and clinical evidence continues to expand, which positions electroceuticals to move from adjunctive interventions to foundational components of next-generation therapeutic strategies. By leveraging the inherent bioelectricity of living systems, electroceuticals offer a unifying approach to controlling infection, restoring immune balance, and enabling tissue regeneration addressing some of the most persistent challenges in modern medicine through fundamentally different means.
